# A Comparison of the Convenience, Quality of Interaction, and Satisfaction of Virtual and In-Person Healthcare Consultations: A Nationwide Study

**DOI:** 10.3390/jcm13175203

**Published:** 2024-09-02

**Authors:** Saad Mohammed AlShareef, Abdullah Abdulaziz AlWabel

**Affiliations:** 1 Department of Medicine, College of Medicine, Imam Mohammad Ibn Saud Islamic University (IMSIU), P.O. Box 7544, Riyadh 13317, Saudi Arabia; 2 Seha Virtual Hospital, Ministry of Health, Riyadh 12382, Saudi Arabia; aaalwabel@moh.gov.sa; 3 King Saud University Medical City, King Saud University, P.O. Box 7805, Riyadh 11472, Saudi Arabia

**Keywords:** Kingdom of Saudi Arabia, satisfaction, telehealth, virtual consultation

## Abstract

**Background**: There are few direct comparisons of service utilization and patient-reported outcomes in patients attending medical consultations in person or virtually. This was a prospective, cross-sectional study of adults engaging with a healthcare practitioner via virtual or in-person consultations. **Methods**: Participants were recruited in person by convenience sampling between November 2023 and January 2024 across Saudi Arabia, and data were gathered on (i) basic demographic and consultation information and (ii) convenience, quality of interaction, and satisfaction with their consultations. **Results**: Of 3196 individuals who completed the survey, 28.7% had attended their most recent healthcare interaction virtually and 71.3% had attended in person. Participants attending virtual consultations were more likely to live rurally (69.0% vs. 21.9% for in-person consultations; *p* < 0.001). Virtual appointments were more common for primary care and diabetes/endocrinology but not surgical specialties (*p* < 0.001), and private apps and hospitals more frequently provided virtual appointments. **Conclusions**: Overall, patients found virtual consultations to be significantly more convenient, prompt, private, and well communicated than in-person appointments, translating into extremely high satisfaction (97.4% overall vs. 84.0% for in-person consultations; *p* < 0.001). This study provides population-level data on the current prevalence of telehealth use in Saudi Arabia. Further prospective research demonstrating the clinical noninferiority of telemedicine could help promote further uptake in specialties such as surgery.

## 1. Introduction

Telehealth uptake in the Middle East has lagged behind many other countries [1]. However, Saudi Arabia, through the delivery of telemedicine via outpatient telemedicine clinics (virtual clinics), 937 call centers, and the Sehhaty smartphone application according to global best practices [2], has consistently advocated, implemented, and developed telemedicine in the country since 1990 [3]. This is important, as about 20% of the population lives in rural areas [4]. Given that smartphone and internet access are nearly universal (>90%) [5,6], the effective implementation of telemedicine can facilitate best healthcare practices in underserved and rural communities [1].

Patients are generally very satisfied with telemedicine [7,8,9,10,11], including in Saudi Arabia [9,10,12,13,14,15,16,17]. Although a recent study showed that over a million virtual consultations were delivered in Saudi Arabia over eighteen months [18], the overall prevalence of telemedicine use is unknown, and there are little direct data on differences in service utilization and patient-reported outcomes of convenience, quality of interaction, and satisfaction between those attending medical consultations in person or virtually. Understanding which populations utilize which services and their preferences is essential to plan quality improvement initiatives and target specific areas for service development.

We therefore conducted a prospective, nationwide cross-sectional survey to compare the demographics, service utilization, and patient-reported outcomes of individuals accessing medial consultations virtually and in person. The null hypothesis was that there would be no differences in demographics, service utilization, and patient-reported outcomes between individuals attending consultations virtually and in person, recognizing that any detected differences could provide evidence for focused quality improvement.

## 2. Materials and Methods

This study is reported according to the STROBE statement for cross-sectional studies [19]. This was a prospective, cross-sectional study of adults aged 18 years or older who could complete a questionnaire. The Institutional Review Board of Imam Mohammad Ibn Saud Islamic University approved the study protocol on 1 September 2023 (reference number 588/2023). All participants provided written, signed informed consent. 

Recruitment was carried out between November 2023 and January 2024 across all regions of Saudi Arabia. Participants were convenience sampled in public areas by 16 medical students trained in the study objectives and the questionnaire, who read each question to participants and recorded the answers to ensure complete data collection. Individual responses were deidentified (from consent forms) for data analysis, and participants were coded using sequential unique identifiers within the analysis spreadsheet. As convenience sampling is inherently biased and relevant only to the study population, we randomly sampled a large population with a similar demographic profile to the wider population. 

The questionnaire is presented in Appendix A. The first 16 questions collected data on basic demographics (e.g., age, sex, area of residence, access to healthcare), the most recent appointment (e.g., who it was for, how long ago, what the consultation was for), and whether the consultation was virtual or on-site. A further nine questions were conceptually based on the Telehealth Usability Questionnaire (TUQ), designed to assess technology implementation and services across the domains of usefulness and convenience, ease of use, effectiveness, reliability, quality of the interaction, and satisfaction [20]. These nine questions were selected for relevance to both in-person and virtual consultations and captured information about the convenience (one question), quality of interaction (three questions), and satisfaction (five questions) of their most recent healthcare consultation using a seven-point Likert scale, where 1 = strongly disagree, 2 = disagree, 3 = somewhat disagree, 4 = neither agree nor disagree, 5 = somewhat agree, 6 = agree, and 7 = strongly agree.

Statistical analyses were performed in SPSS v29 (IBM Statistics, Armonk, NY, USA). Categorical variables are presented as counts and percentages, and age is presented as mean (SD). Responses to questions about opinions on the respondent’s most recent virtual appointment were dichotomized into “agree” (somewhat agree, agree, and strongly agree) or “neutral or disagree” (strongly disagree, disagree, somewhat disagree, and neither agree nor disagree). Associations between variables and these categorizations were assessed with the chi-squared test or Fisher’s exact test for 2 × 2 contingency tables with small expected frequencies (Student’s *t*-test for age). A *p*-value of <0.05 was considered significant.

## 3. Results

Overall, 3196 individuals completed the survey, with an average age of 46.1 (13.7) years; 1627/3196 (50.9%) were female, and 1569/3196 (49.1%) were male. In total, 916/3196 (28.7%) had attended a virtual consultation as their most recent healthcare interaction, while 2280/3196 (71.3%) had attended in person. 

A comparison of participant demographics according to the most recent type of consultation is shown in Table 1. Participants attending virtual consultations were more likely to be female (53.7% vs. 50.9% for in-person consultations; *p* = 0.046), live in rural areas (69.0% vs. 21.9% for in-person consultations; *p* < 0.001), and consequently live further away from their nearest hospital (only 29.7% living within 50 km of their nearest hospital vs. 79.3% living within 50 km for those attending in-person; *p* < 0.001).

There were also differences in the general appointment characteristics of individuals attending virtual or in-person appointments (Table 2). Virtual appointments were more common than in-person appointments for respondents who were attending for themselves rather than a family member (87.8% vs. 61.9%, *p* < 0.001); attending primary care and diabetes/endocrinology appointments (in-person appointments were especially common for emergency medicine and surgical specialties; *p* < 0.001); and those attending with heart disease and diabetes (33.2% vs. 15.1%; *p* < 0.001), with general check-ups most common for in-person appointments.

Virtual appointments were more likely to have been provided by private apps and hospitals rather than publicly funded services, and although the spectrum of healthcare professionals seen was largely similar between groups, those attending virtual appointments were more likely to see a psychologist than those visiting in-person (2.6 vs. 1.1%; *p* < 0.001). More in-person appointments than virtual appointments were scheduled for follow-ups for results. Those attending virtual appointments expressed a strong preference for that type of appointment (92.8%), while for those attending in-person, the preference for virtual or in-person appointments was roughly equally split (44.4% vs. 53.5%, respectively).

Finally, we assessed differences in convenience, quality of interaction, and satisfaction of in-person vs. virtual consultations (Table 3). Overall, patients found virtual consultations to be significantly more convenient (95.6% and 87.4%, respectively), prompt, private, and well communicated than in-person appointments (all *p* < 0.001). These perceptions of the service translated into extremely high satisfaction levels for virtual appointments (97.4% overall), compared with 84.0% for in-person consultations.

## 4. Discussion

This large-scale, population-wide comparison of virtual and in-person appointments reveals that, in Saudi Arabia, virtual healthcare consultations are common (~30% of consultations) and mainly serve a rural community living far from their nearest hospitals. Our analysis suggests that private healthcare services are more likely to offer virtual consultations than public health services and that virtual services are currently not favored in certain disciplines such as emergency medicine and surgery. Although in-person consultations still enjoyed relatively high perceived convenience, interaction, and satisfaction from users (>80% in most cases), participants attending virtual consultations were consistently—and nearly universally—satisfied with the convenience and interactions of their consultations, which translated into extremely high (>95%) satisfaction.

Like in many countries, Saudi Arabia has had a long-term policy on telemedicine use, expansion, and enhancement that predated the COVID-19 pandemic [3]. Although there are little data from Saudi Arabia, before the pandemic, one study on the prevalence of telehealth use in Gulf Cooperation Council (GCC) countries reported that only about 11% of respondents were exposed to telehealth before the COVID-19 pandemic [21], which increased over 250% during the pandemic to about 40% of all users. Assuming parity within the GCC, our prevalence data (28.7% attending a virtual consultation) suggest that at least some of the effects of the pandemic on telemedicine use may have persisted in Saudi Arabia. Indeed, in their 2023 study, Almalki et al. [22] reported that about a quarter of participants attending primary health centers in Riyadh utilized telemedicine. Mirroring these findings, Al-Rayes et al. [23] reported that both awareness and utilization of the 937-Telephone Health Services—a free, 24/7 confidential telephone service that provides medical and administrative health care services, increased from 46% and 42% before the pandemic to 66% and 78% during the pandemic, respectively. Although still much lower than telehealth utilization in Western countries (e.g., ~40% in the United States in the post-COVID-19 era) [24,25,26], our data suggest that Saudi Arabia has progressed in terms of meeting its vision of improving healthcare service accessibility through telehealth across the Kingdom following Saudi Vision 2030 [27]. This progress may have at least in part been driven by the need to control infection during the pandemic.

One of the largest potential advantages of telehealth use is its suggested benefits in increasing access to care and reducing health disparities in specific populations, such as rural and underserved communities. However, to date, there has been little evidence that telehealth preferentially serves these communities. For example, previous reports from Canada [28], the United States [24,29], and Saudi Arabia [18,22] have reported either no difference or increased utilization of virtual health services in urban, rather than rural, locations. Although it has been suggested that access to technology may be lower in rural areas, coupled with cultural factors and a preference for in-person consultations, which may be barriers to telehealth in rural settings [30], our data show a promising uptake of virtual consultations in individuals living in rural locations without close access to hospitals. This discrepancy with previous findings hopefully reflects a genuine shift in healthcare utilization towards telehealth use in individuals in rural settings living distant from secondary and tertiary services and the realization of the promise of telehealth to overcome the barrier of the inconvenience and cost of traveling to healthcare appointments in these settings [14]. 

Our finding that virtual appointments were more common than in-person appointments for respondents attending primary care and diabetes/endocrinology appointments is consistent with previous data showing variable telehealth utilization across specialties but very low utilization for surgical visits and high utilization for endocrinology clinic visits [29,31], as well as high utilization within primary care in Saudi Arabia [18]. Similarly, relatively higher virtual consultation use by those seeing a psychologist than those visiting in person is consistent with previous data showing high telemedicine use by mental health professionals [31]. While virtual medicine may be truly inappropriate for emergencies requiring urgent intervention, there is plenty of evidence that telemedicine could play an active role in surgical care and surgical specialties, especially in the specific scenario of regular telemedicine for postoperative follow-up [32]. It is important to highlight opportunities for surgeons to utilize telemedicine to optimize their practice. Where telemedicine services do not exist or there is resistance to their implementation, there is a need for high-quality, prospective implementation science research to prove the clinical noninferiority of telehealth for outcomes of interest while maintaining, or even improving, patient satisfaction. For instance, Mariani et al. [33] performed a head-to-head prospective comparison of the feasibility and effectiveness of virtual visits compared with in-person visits for patients requiring clinical electrophysiology evaluation and found no significant differences between the two consultation types in terms of symptoms, remote monitoring alerts, and urgent hospitalizations between groups. This was coupled with an increase in satisfaction for patients receiving virtual appointments [33]. In our opinion, providing a sound, objective evidence base through the implementation science framework—which also takes contextual barriers and health economics into account [34]—provides the best route to changing standards of care.

It was interesting to note that virtual appointments were more likely to have been provided by private apps and hospitals rather than publicly funded services, perhaps reflecting different attitudes to healthcare expenditure within the private and public healthcare systems. Nevertheless, although telehealth is often assumed to be a cost-effective means to deliver healthcare [35], it is worth remembering that the implementation of telehealth and encouraging service use must be driven by clinical needs and benefits. In fact, when added to traditional services, telehealth may increase costs [35]. Nevertheless, a recent meta-analysis showed that telemedicine is associated with very high patient satisfaction [8]. The very high convenience and satisfaction levels reported by our participants are similar to previous findings from Saudi Arabia, where >80% of survey respondents were either very satisfied or satisfied with the overall quality of care and telemedicine experience in both general [9,10,12,13,14] and specialist [15,16,17] settings. Our direct head-to-head comparison now adds further weight to the evidence that telemedicine not only delivers comparable quality and outcomes to traditional in-person visits [36] and more efficient appointments but also extremely high levels of satisfaction.

This study has some limitations. We used a convenience sampling methodology, which of course may not be representative of the population as a whole. Nevertheless, to mitigate against unintended bias, we sampled a large cohort of individuals with a similar demographic profile as the wider population. Confirming this, the demographic profile reflected the relatively young population of Saudi Arabia and approximately four-fifths living in urban areas [4,37], increasing confidence that the survey is representative of the wider population and is therefore generalizable. Although there can be recall bias in survey studies, the majority of appointments were within the preceding few months.

Despite the growing body of evidence that virtual medicine is associated with high levels of patient satisfaction, it remains unclear overall whether it offers good value for money [35] or whether it has other impacts on the healthcare team. Encouragingly, a recent meta-analysis suggested that, overall, physicians are satisfied with telehealth for both patient care and consultations with other physicians [38]. It is also unclear whether it marginalizes certain groups or widens disparities. Any prospective studies to prove the clinical equivalence of virtual and in-person consultations must be supported by health economic analyses and account for social determinants of health. Management must also have a visionary policy to ensure equitable service delivery.

## 5. Conclusions

This is one of the largest studies conducted to date comparing virtual and in-person healthcare interactions, and our findings provide population-level data on the current prevalence of telehealth use in Saudi Arabia (~30% of consultations). Although telehealth is often touted as a solution for bridging inequality, not least in serving rural areas, data supporting uptake in rural areas have been lacking. Our data now suggest that Saudi Arabia has progressed in terms of meeting its vision of improving healthcare service accessibility. Virtual consultations are associated with extremely high levels of perceived convenience, quality of interaction, and satisfaction. Although not currently used by specific specialties, further education and awareness of the benefits of telemedicine—supported by high-quality implementation studies to provide objective evidence of clinical noninferiority—could help promote further uptake in specialties such as surgery.

## Figures and Tables

**Table 1:** A comparison of participant demographics between patients attending virtual or in-person consultations.

		Virtual		In-Person		
Characteristic		Number	%	Number	%	*p*-Value
Sex	Male	424	46.3	1145	50.2	0.046
	Female	492	53.7	1135	50.9	
Age (mean, SD)		47.2, 14.1		45.7, 13.6		0.003
Relationship status	Married	667	72.8	1640	72.2	0.631
	Not married	249	27.3	640	28.1	
Area of residence	Central	356	38.9	1092	47.9	<0.001
	Eastern	148	16.2	256	11.2	
	Northern	116	12.7	208	9.1	
	Southern	104	11.4	172	7.5	
	Western	192	21.0	552	24.2	
Urban or rural	Urban	284	31.0	1780	78.1	<0.001
	Rural	632	69.0	500	21.9	
Distance from nearest hospital	<50 km	272	29.7	1808	79.3	<0.001
	50–100 km	108	11.8	140	6.1	
	100–300 km	268	29.3	148	6.5	
	>300 km	248	27.1	96	4.2	
	Unsure	20	2.2	88	3.9	

**Table 2:** A comparison of general appointment characteristics between patients attending virtual or in-person consultations.

		Virtual		In-Person		
Characteristic		Number	%	Number	%	*p*-Value
Time since most recent appointment	<3 months	436	47.6	1412	61.9	<0.001
	3–6 months	332	36.2	440	19.3	
	7–9 months	76	8.3	136	6.0	
	10–12 months	48	5.2	64	2.8	
	>12 months	24	2.6	228	10.0	
Appointment patient	Participant	804	87.8	1848	81.1	<0.001
	Someone else (e.g., child, family member)	112	12.2	432	18.9	
Department	Allergy and immunology	0	0.0	24	1.1	<0.001
	Cardiology	44	4.8	163	7.1	
	Dermatology	8	0.9	92	4.0	
	Diabetes and endocrinology	116	12.7	165	7.2	
	Emergency	0	0.0	72	3.2	
	ENT	40	4.4	156	6.8	
	Gastroenterology	20	2.2	84	3.7	
	General surgery	0	0	72	3.2	
	Hematology	0	0.0	12	0.5	
	Infectious diseases	0	0.0	4	0.2	
	Nephrology	24	2.6	52	2.3	
	Neurology	60	6.6	102	4.5	
	Obstetrics and gynecology	12	1.3	76	3.3	
	Oncology	0	0.0	16	0.7	
	Ophthalmology	4	0.4	48	2.1	
	Pediatrics	36	3.9	72	3.2	
	Primary care	412	45.0	894	39.2	
	Psychiatry	68	7.4	36	1.6	
	Respiratory medicine	40	4.4	36	1.6	
	Rheumatology	8	0.9	32	1.4	
	Sleep medicine	8	0.8	8	0.4	
	Smoking cessation	12	1.3	12	0.5	
	Urology	4	0.4	48	2.1	
Reason for attendance	Allergy (including asthma)	56	6.1	101	4.4	<0.001
	Arthritis, joint and back pain	16	1.7	164	7.2	
	Neurology, including headaches	28	3.1	44	1.9	
	Respiratory problems (excluding asthma)	80	8.7	192	8.4	
	Psychological or psychiatric conditions	52	5.7	52	2.3	
	Cardiovascular disease, including diabetes	304	33.2	344	15.1	
	Dermatological conditions	16	1.7	62	2.7	
	Pediatrics	8	0.9	4	0.2	
	Gastrointestinal conditions	16	1.7	16	0.7	
	Sleep problems, including OSA	56	6.1	45	101	
	Obesity	8	0.9	13	0.6	
	Other, including general health check-up or smoking cessation	260	28.4	1218	53.4	
	Peri- or postnatal care	8	0.9	24	1.1	
	Renal	8	0.9	0	0.0	
Who provided the appointment?	Call 973	28	3.1	32	1.4	<0.001
	Other government hospital	344	37.6	1056	46.3	
	Other private healthcare app	72	7.9	12	0.5	
	Private hospital	324	35.4	692	30.4	
	Seha virtual hospital	32	3.5	28	1.2	
	Sehhaty app	116	12.7	460	20.2	
Healthcare professional seen	Doctor	876	95.6	2160	94.7	<0.001
	Nurse	8	0.9	32	1.4	
	Psychologist	24	2.6	24	1.1	
	Don’t know	8	0.9	64	2.8	
New appointment or for pre-existing condition	New consultation	376	41.0	788	34.6	<0.001
	Routine follow-up	360	39.3	796	34.9	
	Follow-up for results	56	6.1	448	19.6	
	Follow-up for medication refill	120	13.1	236	10.4	
	Missing	4	0.4	12	0.5	
Preferred type of appointment	In-person	40	4.4	1220	53.5	<0.001
	Virtual	848	92.8	1012	44.4	
	Missing	28	3.1	48	2.1	

**Table 3:** Convenience, interaction, and satisfaction of in-person vs. virtual consultations.

Question		Virtual		In-Person		*p*-Value
		Number	%	Number	%	
Overall, I found my last consultation very convenient	Neutral or disagree	28	3.1	288	12.6	<0.001
	Agree	888	95.6	1992	87.4	
My consultation started on time	Neutral or disagree	44	4.8	460	20.2	<0.001
	Agree	872	95.2	1820	79.8	
My privacy was respected	Neutral or disagree	32	3.5	200	8.8	<0.001
	Agree	884	96.5	2080	91.2	
My healthcare provider explained things in a way that was easy to understand	Neutral or disagree	32	3.5	240	10.5	<0.001
	Agree	884	96.5	2040	89.5	
I felt comfortable communicating with the clinician during my consultation	Neutral or disagree	32	3.5	276	12.1	<0.001
	Agree	884	96.5	2004	87.9	
This type of consultation is an acceptable way to receive healthcare services	Neutral or disagree	32	3.5	172	7.5	<0.001
	Agree	884	96.5	2108	92.5	
I would use this type of consultation service again	Neutral or disagree	28	3.1	420	18.4	<0.001
	Agree	888	96.9	1860	81.6	
I would recommend this type of consultation to family and friends	Neutral or disagree	32	3.5	452	19.8	<0.001
	Agree	884	96.5	1828	80.2	
Overall, I was satisfied with this type of consultation	Neutral or disagree	24	2.6	364	16.0	<0.001
	Agree	892	97.4	1916	84.0	

## Data Availability

All raw data are available from the corresponding author upon reasonable request.

## Appendix A

**Questionnaire on the use, convenience, effectiveness, reliability, and satisfaction of healthcare consultations in Saudi Arabia**

1. Are you male or female?

Options: male, female

2. How old are you?

Option: free text, number

3. Are you married/widowed, separated/divorced, never married?

Options: married/widowed, separated/divorced, never married

4. Which part of the country do you live in?

Options: central/eastern/western/northern/southern

5. Do you live in a city or in the countryside?

Options: city, countryside

6. How far are you from your nearest hospital?

(<50 km, 50–100 km, 100–300 km, > 300 km, I don’t know)

7. Approximately how long ago was the appointment (in months)?

Option: less than three months, from 3–6 months, from 7–9 months, from 10–12 months, more than a year and after a period of COVID-19 restrictions, during a period of COVID-19 restrictions.

8. Was the appointment for you or with someone else (i.e., a child, family member)

Options: for myself, for someone else

9. Which department was your appointment with?

Options: primary care (family/general medicine), medicine (cardiology, respiratory medicine, nephrology, diabetes and endocrinology, allergy and immunology, neurology, rheumatology, infectious disease, hematology, oncology, gastroenterology, psychiatry, psychology and psychotherapy, smoking cession, dermatology, sleep medicine), surgery (general surgery, ENT, ophthalmology, urology, orthopedics), pediatrics, obstetrics and gynecology, emergency

10. Why did you see the healthcare professional?

Options: hypertension, hyperlipidemia, arthritis & joint disorders, diabetes, depression or anxiety, obesity, asthma, allergic rhinitis and or allergic sinusitis, cancer, COPD, osteoporosis, skin disorders, back problems, upper respiratory infections, prenatal or post-natal care, chronic neurologic disorders, headaches and migraines, GERD, irritable bowel syndrome, obstructive sleep apnea, insomnia, other sleep disorder, psychotherapy, smoking cessation, periodic health examination, other (free text)

11. Was this your first consultation for this complaint? Options: yes/no

12. Who provided the appointment?

Options: Call 973, Sehhaty app, Seha virtual hospital, other government hospital, private hospital, Other private healthcare apps like Cura, Vezeeta, labayh. etc., I can’t remember.

13. What type of healthcare professional did you see?

Options: doctor, nurse, pharmacist, physiotherapist, psychologist, occupational therapist, can’t remember/don’t know

14. Was your appointment a new consultation or for a pre-existing health problem?

Options: new consultation, routine follow-up for ongoing health problem, follow-up for results, follow-up for medication re-fill

15. For healthcare consultations, which type you prefer?

Virtual consultation, onsite consultation

16. Thinking back on the last consultation, was it virtual of onsite consultation?

**ALL THE FOLLOWING ARE ON A 7-POINT LIKERT SCALE WHERE 1—STRONGLY DISAGREE, 2—DISAGREE, 3—SOMEWHAT DISAGREE, 4—NEITHER AGREE NOR DISAGREE, 5—SOMEWHAT AGREE, 6—AGREE, 7—STRONGLY AGREE, N/A**

17. Overall, I found my last consultation very convenient **(usefulness/convenience)**

18. My last consultation visit started on time **(interaction)**

19. My privacy was respected **(interaction)**

20. My healthcare provider explained things in a way that was easy to understand **(interaction)**

21. I felt comfortable communicating with the healthcare professional **(satisfaction)**

22. This type of consultation was an acceptable way to receive healthcare services **(satisfaction)**

23. I would use this type of consultation services again **(satisfaction)**

24. I would recommend this type of consultation to family and friends **(satisfaction)**

25. Overall, I was satisfied with this consultation **(satisfaction)**
